# Experimental Analysis of Handcart Pushing and Pulling Safety in an Industrial Environment by Using IoT Force and EMG Sensors: Relationship with Operators’ Psychological Status and Pain Syndromes

**DOI:** 10.3390/s22197467

**Published:** 2022-10-01

**Authors:** Milos Petrovic, Arso M. Vukicevic, Marko Djapan, Aleksandar Peulic, Milos Jovicic, Nikola Mijailovic, Petar Milovanovic, Mirko Grajic, Marija Savkovic, Carlo Caiazzo, Velibor Isailovic, Ivan Macuzic, Kosta Jovanovic

**Affiliations:** 1 School of Electrical Engineering, University of Belgrade, Bulevar kralja Aleksandra 73, 11000 Belgrade, Serbia; 2 Faculty of Engineering, University of Kragujevac, Sestre Janjic 6, 34000 Kragujevac, Serbia; 3 Faculty of Geography, University of Belgrade, Studentski trg 3, 11000 Belgrade, Serbia; 4 The AI Institute of Serbia, Fruškogorska 1, 21000 Novi Sad, Serbia; 5 Center of Bone Biology, Faculty of Medicine, University of Belgrade, Dr Subotica 4/2, 11000 Belgrade, Serbia; 6 Laboratory of Bone Biology and Bioanthropology, Faculty of Medicine, Institute of Anatomy, University of Belgrade, Dr Subotica 4/2, 11000 Belgrade, Serbia; 7 Center for Physical Medicine and Rehabilitation, Faculty of Medicine, University Clinical Center of Serbia, University of Belgrade, Pasterova 2, 11000 Belgrade, Serbia

**Keywords:** push and pull, forces, EMG, psychological status, pain syndromes

## Abstract

Non-ergonomic execution of repetitive physical tasks represents a major cause of work-related musculoskeletal disorders (WMSD). This study was focused on the pushing and pulling (P&P) of an industrial handcart (which is a generic physical task present across many industries), with the aim to investigate the dependence of P&P execution on the operators’ psychological status and the presence of pain syndromes of the upper limbs and spine. The developed acquisition system integrated two three-axis force sensors (placed on the left and right arm) and six electromyography (EMG) electrodes (placed on the chest, back, and hand flexor muscles). The conducted experiment involved two groups of participants (with and without increased psychological scores and pain syndromes). Ten force parameters (for both left and right side), one EMG parameter (for three different muscles, both left and right side), and two time-domain parameters were extracted from the acquired signals. Data analysis showed intergroup differences in the examined parameters, especially in force integral values and EMG mean absolute values. To the best of our knowledge, this is the first study that evaluated the composite effects of pain syndromes, spine mobility, and psychological status of the participants on the execution of P&P tasks—concluding that they have a significant impact on the P&P task execution and potentially on the risk of WMSD. The future work will be directed towards the development of a personalized risk assessment system by considering more muscle groups, supplementary data derived from operators’ poses (extracted with computer vision algorithms), and cognitive parameters (extracted with EEG sensors).

## 1. Introduction

With the ongoing progress of technology, workplace safety standards have also risen, proposing a “zero injuries” goal as an acceptable number of workplace accidents [1]. This challenge is studied by safety science and ergonomics, scientific disciplines that aim to design and/or improve workplaces through the minimization of discomfort, exertion, stress, and elimination of hazards and risk of injuries [2]. Studying how to improve the safety of repetitive tasks still remains highly challenging and relevant since its non-ergonomic execution represents a major cause of work-related musculoskeletal disorders (WMSD) [3]. WMSDs are described as a long-term accumulation of negative effects caused by repetitive unsafe acts (manual handling, heavy physical jobs, and awkward postures) [4]. Moreover, they have multiple negative sociological and economic implications. In the European Union in 2007, 8.6% of the people aged 15 to 64 that worked or previously worked reported a work-related health problem, which corresponds to approximately 23 million persons with WMSDs as the main work-related health problem (60%) [5]. This study is restricted to analyzing the task of P&P handcarts, which was chosen as a representative dynamic physical task whose variants are present across many industries (including warehouses, transportation, healthcare, sports, etc.).

Chen et al. reconstructed the human model in order to obtain the visualization of the WMSD risk factors of, among the others, the P&P task using a wearable and connected gait analytics system and the Kinect [6]. Aiming to determine ergonomic risk levels of P&P, the construction worker tasks were studied using a wearable insole pressure system [7]. Gómez applied a statistical model for the prediction of work-related musculoskeletal discomfort and ergonomic evaluation to data collected by means of direct observation and surveys as a part of the meat processing industry (P&P of heavy loads included) [8]. Nath et al. created a machine learning-based solution for data acquisition and data processing, human activity recognition of three classes (category 0: wait; category 1: lift/lower/carry; category 2: push/pull), estimation of activity durations and frequencies, and assessment of overexertion and ergonomic risks using time-stamped motion data from a body-mounted smartphone [9].

The most useful information about the muscular load of the industrial worker and the potential threat of WMSDs could be provided by electromyography (EMG) data, which measures the electrical activity of the skeletal muscle in response to nerve stimulation. The idea of analyzing EMG data in the industrial workplace dates back to 1990 [10], and detailed instructions for measurement methodology have already been presented [11]. With the aim of analyzing the impact of a particular task on the human body and reducing its physical demands, previous studies focused on the framework for adaptation of the robot to human fatigue in different human–robot collaborative industrial tasks [12]. EMG electrodes were used in a specially designed laboratory experiment simulating roofing jobs to examine the effects of common risk factors based on lower back muscle activity and working frequency [13]. Salas et al. conducted a comparative analysis of experienced and inexperienced rodworkers using EMG and the Xsens MTx Xbus system to evaluate levels of back moments L4/L5 and time spent in upright and flexed posture [14]. Peppoloni et al. proposed a wireless wearable system (using IMU sensors and EMG) for the assessment of WMSD risks for the upper limb performing the task of repetitive object lifting and dropping [15].

Safe performance of work activities, especially repetitive ones, requires the worker’s continuous attention. Given that attention is affected by a person’s psychological/emotional status, it is expected that individuals with a more negative psychological status (anxiety, stress, depression, or apathy) would be at a higher risk of workplace-related injury. There are still scarce data on the association between psychological status and the risk of workplace injury. In general, depression is associated with concentration difficulties, and it has been shown that chronic stress negatively affects an individual’s attention and executive function [16]. Depression and anxiety increase the risk of injury among athletes [17], and workers perceive that symptoms of anxiety and depression place them at risk of workplace injury [18]. A recent study has examined longitudinal associations between depression or anxiety with a work-related injury and showed that the presence of depression was able to predict injury, while the effect of anxiety was less clear [19]. However, none of the previous studies have considered the association of psychological status and patterns of activities related to specific tasks such as cart pushing/pulling.

Given that P&P is highly associated with risks from musculoskeletal injuries, there is interest in the effects of various aspects of the pushing/pulling task on loads experienced by the body, especially the spine [20]. However, while cart P&P tasks might affect the spine and extremities, it is also reasonable to assume that the presence of pain syndromes of the upper extremities as well as the pain syndromes and reduced mobility of the spine would affect how an individual performs the pushing/pulling task. A previous study showed that upper limb pain slows down force generation at handgrip [21]. In addition, the effect of handle stability on maximum push/pull force was investigated to enhance individuals’ push/pull capabilities and reduce fatigue and musculoskeletal disorders [22]. Nevertheless, previous studies have not considered the actual performance of individuals with different states of musculoskeletal health.

As may be noted, P&P safety risks could be divided into (1) ergonomics (workplace-related) and (2) individual (poor health habits or physical conditions) [23]. While the first topic is well studied in the literature, recent studies have started paying more attention to the studying correlation between WMSDs and individual operator characteristics and/or behavior in the workplace [24,25]. In this study, we aim to analyze in detail P&P safety by enveloping the above-mentioned aspects.

## 2. Materials and Methods

### 2.1. Study Participants

The study was approved by the Ethics Committee of the Faculty of Medicine, University of Belgrade, and all of the included participants provided informed consent for their participation in the study. A total of 20 male individuals (mean age: 35.0 ± 5.8 years) were included in the study (Table 1). The mean body weight was 94.9 ± 11.0 kg, and the mean height was 186.0 ± 8.8 cm. None of the subjects had previous workplace injuries. The participants were initially selected from the groups of experienced and nonexperienced in physical tasks in industry, with various levels of subjectively reported WMSDs (primarily back pain). Such inclusion criteria were chosen to enable investigation of multiple research problems upon the initial analysis of the acquired data. After the physical and psychological assessments were performed by clinical professionals, the aim was to examine the correlation between the clinical examinations and patterns in execution of physical tasks such as P&P.

### 2.2. Physical and Psychological Assessment

We used the DASS, a 42-item self-report instrument designed to measure negative emotional states including depression (dysphoria, hopelessness, devaluation of life, self-deprecation, lack of interest/involvement, anhedonia, and inertia), anxiety (autonomic arousal, skeletal muscle effects, situational anxiety, and subjective experience of anxious affect), and tension/stress (chronic non-specific arousal, including difficulty, relaxing, nervous arousal, and being easily upset/agitated, irritable/over-reactive, and impatient) [26,27]. The Serbian adaptation [28] of the instrument was used from the official website of the DASS scale. Each of the three DASS scales contains 14 items, and the participants were asked to rate each item on a 4-point Likert scale to show how severely or frequently they experienced each state over the past week. Then, we calculated the scores for Depression, Anxiety, and Stress by summing the scores for the relevant items. In accordance with the DASS manual, the threshold scores for Depression, Anxiety, and Stress were 15, 8, and 10, respectively. Apathy was evaluated by the Starkstein apathy scale [29,30,31]. It contains 14 items, and the total score can be in the range between 0 and 42. Scores equal to or higher than 14 were considered clinically meaningful apathy.

A standard physical examination was performed by a senior specialist in physical medicine and rehabilitation. Particular emphasis was placed on pain syndromes of the upper extremities (pain on palpation of the trigger points of the target muscles), assessment of a range of motion of the spine, and signs of a radicular lesion (straight leg raise test). Pain syndromes were scored on a Verbal Rating Scale (VRS) from 0 to 4, where 0 indicated ‘no pain’ and 4 indicated ‘very severe pain.’

### 2.3. Experiment Design

**Figure 1:**
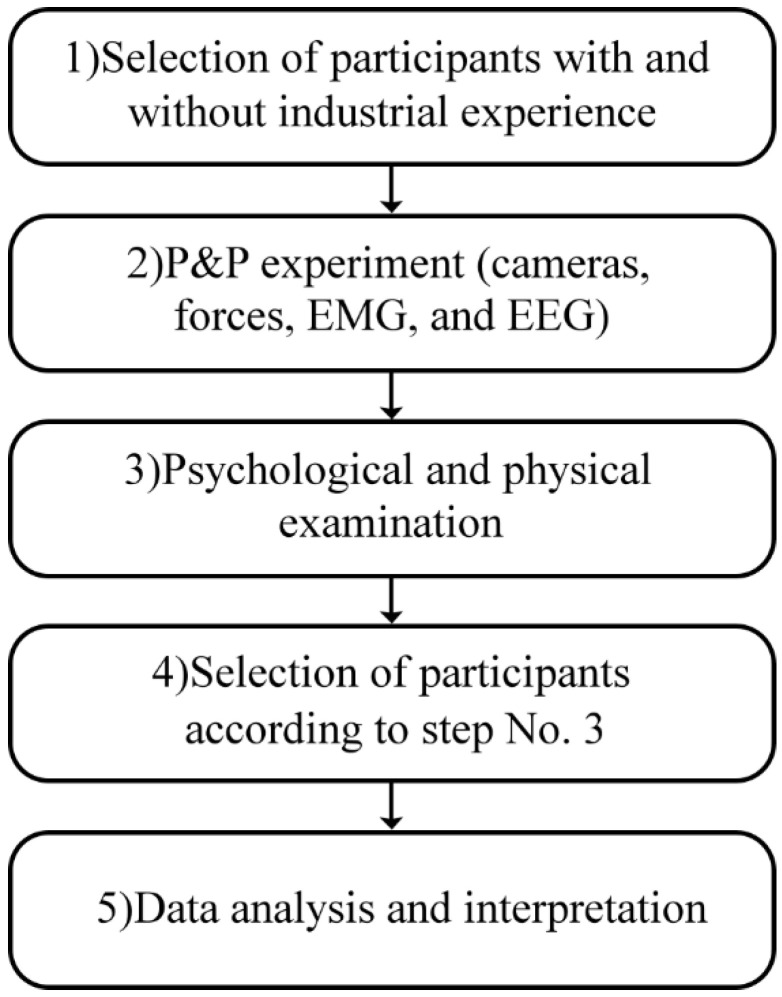
Flowchart of the experiment.

**Figure 2:**
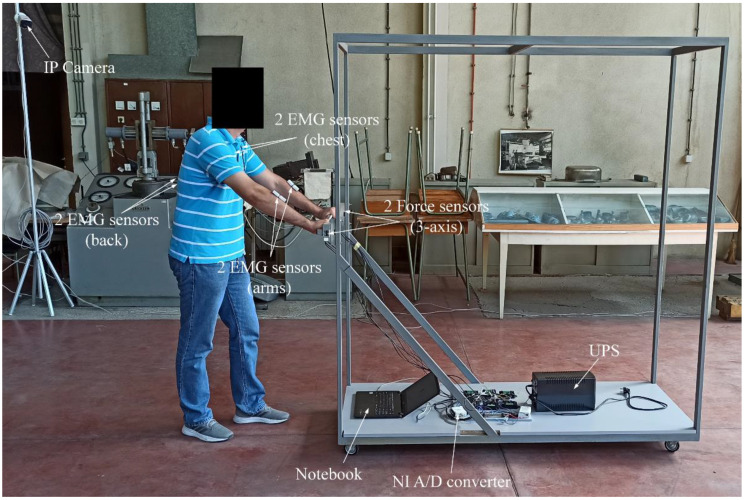
Trolley and equipment used in the experiment.

The flowchart of the experiment is presented in Figure 1. The industrial trolley was made of steel-welded thin-walled box profiles (2 × 4 cm). The dimensions of the industrial trolley are 180 × 73 × 195 (L × W × H). The total height of the trolley with wheels is 205 cm. The aluminum handle, ergonomically shaped, is placed at 115 cm from the floor. There are four rubberized wheels and their size is ⍉75. The approximate weight of the industrial trolley is 100 kg (Figure 2).

**Figure 3:**
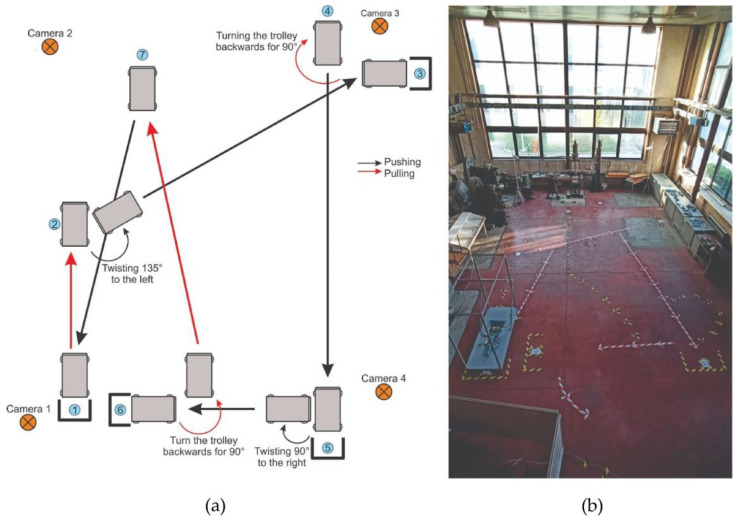
Considered P&P tasks and experimental environment. (**a**) P&P path sketch and (**b**) Workplace polygon.

The polygon was 9 × 6m in size (Figure 3). The floor was flat, similar to the industrial one, with occasional transitions from one type of base to another. This way of manipulating industrial carts (pushing, pulling, and turning) is a typical way of manipulation in industry. It contains all the elements necessary for conducting the experiment in terms of the direction of P&P, and the representation of the rotation of the cart in all directions, bearing in mind the most characteristic movements that occur in the industry.

Participants had to cover a distance of about 30 m in one attempt. The movement was carried out in 5 series, and each series consisted of two attempts (10 repetitions). There was a 2-s pause between the two attempts (with the arms relaxed next to the body). There was a 20-s break between the two series. If a participant had felt any pain or fatigue, the experiment was stopped. The total average time during the whole session was about 11 min per participant.

**Figure 4:**
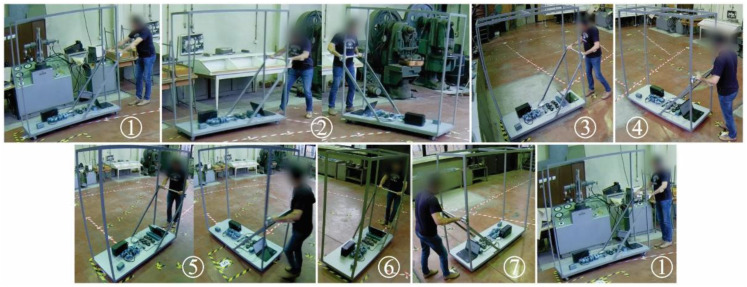
Trolleys’ and operators’ positions during sessions illustrated in Figure 3.

The P&P task is visually presented in Figure 3 and Figure 4. It starts from position ① by pulling the cart to position ②. Then, by turning the cart at the waist 135 degrees to the left, they push to position ③, where it stops at the marked place. Then, the trolley is pulled back and turned backward by 90 degrees to the ④ position. The trolley is pushed to the ⑤ position, stops, and turns at the waist 90 degrees to the left. The trolley is then pushed to position ⑥. By walking backward, the trolley is pulled to position ⑦ and then pushed to the starting position ①.

### 2.4. Data Acquisition

The P&P forces were measured using the three-axis force sensors. The Forsentek 3-axis force sensors, on strain gauge technology based on high precision amplified circuits, are connected to the central microprocessor edge device. All signals were sampled at 100 Hz. The measurement hardware includes two identical 3-axis force sensors for the left and right arm each. Based on these measurements, sensor values are processed directly on the edge device. Every sample contains *xR, yR, zR, xL, yL*, and *zL* force values for the right (*R*) and left (*L*) arm. The force intensity *FR* and *FL* are calculated by using equations

(1) $$FR=\sqrt{f_{xR}^{2}+f_{yR}^{2}+f_{zR}^{2}}\;FL=\sqrt{f_{xL}^{2}+f_{yL}^{2}+f_{zL}^{2}}\;.$$

Six EMG surface electrodes were placed on the subject’s body on the latissimus dorsi, pectoralis major, and flexor carpi ulnaris muscles bilaterally (Figure 2). The EMG signals were acquired using the Delsys Trigno Wireless Biofeedback system and the biosignalsplux muscleBAN to measure muscle activity as a reference to the effective physical effort required for the tasks. The Trigno EMG System is supported by EMGworks Acquisition software for standardized and simple data acquisition and EMGworks Analysis software to analyze the recorded signals. Prior to the experiment, the maximal voluntary contraction (MVC) for each muscle was extracted according to the MVC procedures [32]. The MVC represents the maximum value of the EMG signal during the maximum contraction of the specified muscle. Afterward, the recorded EMG signals were filtered and normalized to the corresponding MVC.

All the experiments were recorded with four DAHUA IPC-HFW2831TP-ZS 8MP WDR IR Bullet IP Cameras, with a DAHUA PFS3010-8ET-96 8port Fast Ethernet PoE switch. The host PC had a CPU 1151 INTEL Core i3-8100 3.6GHz 6MB BOX. Cameras were placed in the corners of the workplace (Figure 2), capturing the full body of the participant during the whole experiment.

Before every new participant’s session, we assured that IP cameras were capturing the complete workplace and that there were no dead angles and occlusions, so the participant was visible on every camera throughout the experiment. Each participant had a trial of a few minutes to practice. At the beginning of the experiment, the subject was at position ①. When EMG and force sensors were turned on, supervisors instructed the participant to start. This way, video data, force signals, and muscle signals were recorded almost simultaneously, which eased the process of post-processing.

Based on the psychological assessment and physical examination (Table 2), we identified two extreme groups of participants. Group 1 (two individuals) comprised individuals without high psychological scores of stress, depression, anxiety, and apathy; without pain syndromes of the upper extremity; and without limited mobility of the thoracolumbar spine. Group 2 comprised individuals (three individuals) with two high psychological scores (stress, depression, anxiety, or apathy); with pain syndromes of the upper extremity (at least two active (painful) trigger points with a VRS score of 3 or 4 on palpation); and with limited mobility of the thoracolumbar spine. None of the participants had a positive straight leg raise test. For further analysis, we selected these two groups of participants.

**Figure 5:**
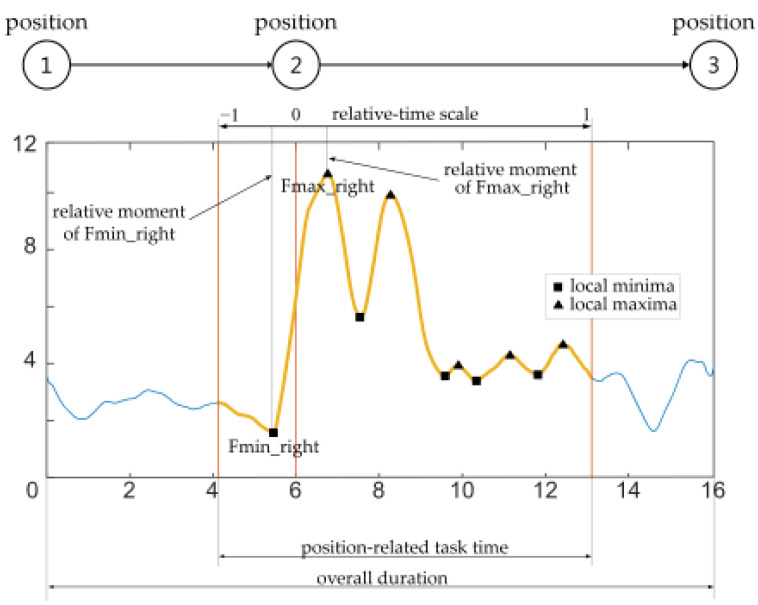
Parameters extracted from the acquired force signal.

For the purpose of data interpretation and analysis, the experiment (Figure 3) was divided into six phases: from position ① to position ③ (P1-2-3); from position ② to position ④ (P2-3-4); from position ③ to position ⑤ (P3-4-5); from position ④ to position ⑥ (P4-5-6); from position ⑤ to position ⑦ (P5-6-7); and from position ⑥ to position ① (P6-7-1). Furthermore, to be able to relate the physical action with the actual position more precisely (e.g., P2), all phases were further divided as follows: the last 30% of the time needed to reach the middle position from the beginning position (e.g., P1-2), and the first 70% of the time from the middle position to the end position (e.g., P2-3), as depicted in Figure 5. All force and EMG parameters were calculated on the aforementioned six sub-segments. Parameters for relative moments of the events of sub-segments were in the range from −1 (30% left of the middle point, e.g., P1) to 1 (70% right of the middle point, e.g., P3). The middle point had a value of 0 (e.g., P2). The force parameters considered for both left and right sensors were: mean value, standard deviation, maximal value, minimal value, the integral value (area under force curve), overall duration, the relative moment of the middle position, the relative moment of the maximal value, the relative moment of minimal value, the maximal difference of consecutive extremums, number of local maxima, number of local minima, and position-related task time. The normalized EMG parameter considered for each muscle was mean absolute value (MAV).

## 3. Results

Table 2 shows the results of force measurements in Groups 1 and 2, as well as the percent of the difference between Groups 1 and 2 for each of the measured parameters. Most of the measured parameters were numerically different between the two groups, with the highest percent of the difference between Group 2 and Group 1 recorded for integral right value (almost 90%) and integral left value. We also calculated the percentage of the difference between the last two series and the first two series in order to illustrate the variability between these series and evaluate the temporal evolution of force patterns. Table 3 shows the results of EMG measurements in Groups 1 and 2, as well as the percent of the difference between Groups 1 and 2 for each of the measured parameters.

## 4. Discussion

The vertical force component had the lowest influence on the resultant forces (both FR and FL), which is the consequence of the handcart handle position placed at the right height, according to standards of industrial P&P. As shown in Table 2, Group 2 had almost equal mean values of left and right force sensors, meaning that these participants conducted the P&P tasks more symmetrically and carefully compared with participants in Group 1. In contrast, subjects in Group 1 relied primarily on the dominant arm, which led to asymmetrical task execution (also validated with a higher maximal right than maximal left-hand value). On average, the overall duration of each sub-segment was longer for Group 2, which means that they carried out the task with more caution. The standard deviation of the left and right sensors was quite low and approximately equal for both groups, which is desirable as it implies the repeatability of the experimental measurements. When it comes to the integral value, one of the most important parameters that directly shows the amount of energy spent for a specific task, Group 2 had almost two times larger values (554.118 and 553.0317) compared with Group 1 (292.9955 and 264.0737). Hence, individuals with high scores of stress, depression, anxiety, or apathy, with pain syndromes of the upper extremity, and with limited mobility of the thoracolumbar spine put significantly more effort into the same task execution than individuals from Group 1. Even though the time of the middle position for both groups was almost identical, the relative time-points of the maximal value of Group 1 preceded the moments of the maximal value of Group 2, suggesting their different motion planning and faster decision-making for the upcoming action needed. The number of local maxima for both left and right sides of Group 2 was approximately 20% larger than that for Group 1, suggesting that despite doing the task more watchfully and symmetrically, the subjects from Group 2 had some sudden unnecessary movements.

The EMG results presented in Table 3, precisely MAVs, indicated that the subjects in Group 2 had higher activity in the tested muscles of the chest and back. Moreover, amplitudes of the left and right sides were symmetrical in Group 2, but they used the left chest and left back muscles significantly more than subjects in Group 1 (especially the left back muscle, with an intergroup difference of over 400%). Participants in Group 1 used the right back and the right chest muscles predominantly, implying that there was a difference in muscle activation patterns for the same task between the groups. Muscle activity of the left and right arms was identical within the group but was 40% higher for Group 2 compared with Group 1. However, the variation of almost all parameters between S9–10 and S1–2 was lower in Group 2 than in Group 1. This might be the result of the caution and persistent task execution of participants in Group 2, as well as the result of the reduced duration time of the sessions for Group 1 (the difference between S9–10 and S1–2 is more than 16%).

To the best of our knowledge, this is the first study that evaluated the composite effects of pain syndromes, spine mobility, and psychological status of the participants on the execution of P&P tasks. We showed that the presence of stress, depression, anxiety, or apathy, in combination with pain syndromes and limited spinal mobility, can drastically influence work performance. Previous studies have analyzed risk factors for work-related injuries in various contexts, and some of them addressed the influence of psychological status, such as depression and anxiety, on the risk of injury [19,33,34]. However, no studies have simultaneously considered the effects of psychological status and muscle pain syndromes of the upper extremities on the patterns of task performance in P&P. A systematic review [35] indicated that pushing/pulling tasks might increase upper extremity symptoms in workers, specifically for shoulders, but it is still unclear whether these tasks favor the development of symptoms in the rest of the upper extremity. Moreover, there is insufficient evidence as to whether pain syndromes of the upper extremity alter the task execution. Here, we showed that pain syndromes of the upper extremity, which reflect overuse injury of the muscles and are common musculoskeletal problems both in workers and in the general population, change the activation patterns of the muscle groups during the conducted experiment, which is very important from a clinical point of view. These results together may provide meaningful information about the cause of injury and provide strategy guidelines for proper task execution, thereby leading to a lower number of WMSDs.

However, it should be acknowledged that the current study has some limitations. First, as this was a pilot study, we focused on a limited number of participants. Second, EMG recordings were based just on three muscles, and further studies should consider other muscles of interest, especially other muscles of the upper extremity. Third, due to the small sample size, in this study, we considered a composite effect of psychological status, pain syndromes of the upper extremities, and spine range of motion, which means that we cannot distinguish the individual effects of these three groups of factors. Considering that musculoskeletal injuries predispose to psychological changes, including depression [19,36,37], it is particularly hard to separate the effects of the physical and psychological factors on task performance. That is another reason why we considered it acceptable to examine the composite effect of physical and psychological factors on the performance of P&P tasks.

Overall, our quantitative assessment of the task performance revealed that the two groups showed different patterns of activation and differential manner of task performance. The activities of the individuals from Group 2 were clearly affected by their characteristics as described above. However, it should also be noted that Group 1 is not devoid of any risk of injury because our analysis of video recordings suggested that those subjects were quicker and less careful with the tasks compared with Group 2. Additional work is needed to define specific actionable steps to reduce the risk of injury in both groups; nevertheless, our work is important as it documents a rationale to further explore the individual characteristics as risk factors for a work-related injury, which is essential for a comprehensive and holistic understanding of workplace safety.

In future work, we aim to further analyze P&P and similar tasks by combining available technology in a unique assessment procedure. P&P has shown to be complex task; therefore, there is a need for more extensive and diverse datasets and more muscle groups to be taken into account (dynamic parameters of the human body). While our qualitative analysis of video recordings suggested some differences in how the tasks are performed by different groups, for a broader analysis, the application of computer vision algorithms, such as pose estimation for assessment of whole body pose, could be incorporated. Some critical information about the current psychological state of workers, in particular alertness and attention, should also be examined during experiments, for example, with the use of electroencephalogram measurements.

## 5. Conclusions

Studying the safety of repetitive physical tasks is of great importance as their non-ergonomic execution has been reported as a major cause of work-related musculoskeletal disorders. In this study, we presented a dedicated acquisition system with the purpose of assessing the safety of P&P of an industrial handcart. The system integrated two three-axis force sensors (placed on the left and right arm) and six EMG electrodes (placed on latissimus dorsi, pectoralis major, and flexor carpi ulnaris muscles). The study included a total of 20 male individuals without previous workplace injuries who were instructed to perform P&P over the complex path of ~30 m (ten repetitions). Detailed quantitative analysis was performed in two subgroups, Group 1 (without high psychological scores of stress, depression, anxiety, and apathy; without pain syndromes of the upper extremity; and without limited mobility of the thoracolumbar spine) and Group 2 (with high scores of stress, depression, anxiety, or apathy; with pain syndromes of the upper extremity; and with limited mobility of the thoracolumbar spine). The analysis of the acquired signal included the computation of ten force parameters (for both left and right side), one EMG parameter (for three different muscles, both left and right side), and three time-domain parameters. Out of all the parameters, the force parameter, integral values, and the EMG parameter, MAV of the left latissimus dorsi, were substantially different between the two groups. Specifically, the force parameter was approximately 100% and the EMG parameter was more than 400% higher in Group 2, suggesting that participants in Group 2 put significantly more effort into the same task. Moreover, the MAV of the left and right sides had similar values in Group 2, which was not the case for Group 1, implying different muscle activation patterns and that subjects in Group 2 conducted the task more symmetrically and carefully than those in Group 1. We can conclude that psychological factors and pain syndromes of the upper limb muscles have a significant impact on P&P safety and probably also on the risk of WMSD. Accordingly, future research on this topic will be directed towards considering more industrial workplaces and muscle groups, as well as incorporating computer vision algorithms for pose analysis (or alternative visual systems, such as [38]) and EEG sensor data for monitoring brain activities during working hours [39]. Additionally, innovative methods such as the use of brain–computer interface (BCI), EEG-based attention test with CPT and TOVA, and eye-tracking systems could be helpful to further examine attention levels [40,41,42,43]. To enhance safety, industrial workplaces of the future will rely on the integration of new industry 4.0 technologies: Safety 4.0 [44,45], Quality 4.0 [46], Logistics 4.0 [47], and collaborative robotics, especially from the perspective of ergonomics [48] and neuroergonomics [49].

## Figures and Tables

**Table 1:** Characteristics of the participants.

Participant Number	Age	Body Weight (kg)	Height (cm)	DASS Score Stress	DASS Score Anxiety	DASS Score Depression	Apathy Score	Straight Leg Raise Test +	Reduced Range of Motion of the Thoracolumbar Spine +	Number of Active (Painful) Trigger Points of the Upper Limb #
1	28	110	185	10	0	0	4	0	0	0
2	40	100	202	12	6	1	16 *	0	+	0
3	41	102	188	13	4	4	15 *	0	+	0
4	36	92	190	5	1	0	15 *	0	+	5
5	26	65	163	5	5	8	17 *	0	0	0
6	30	100	194	12	8 *	11 *	11	0	+	4
7	35	92	178	15 *	1	2	15 *	0	+	6
8	37	115	188	13	6	3	16 *	0	0	0
9	52	94	184	8	6	0	10	0	0	0
10	38	90	190	15 *	6	5	14 *	0	0	0
11	35	99	186	11	9 *	4	16 *	0	+	5
12	34	80	175	10	1	1	10	0	0	2
13	33	92	186	16 *	7	4	16 *	0	+	2
14	38	100	190	6	4	2	13	0	+	2
15	33	105	190	9	1	0	13	0	+	0
16	30	97	193	14	8 *	3	16 *	0	0	0
17	29	94	192	8	4	5	14 *	0	+	1
18	30	101	198	10	7	8	17 *	0	0	0
19	34	85	174	16 *	6	4	21 *	0	+	0
20	35	85	187	9	2	1	12	0	0	2

*—above the threshold for psychological scores. #—pain on palpation of the trigger points of the muscles of the upper limb scored on a Verbal Rating Scale (VRS) as 3 (‘severe pain’) and 4 (‘very severe pain’); total number of investigated points was 10.

**Table 2:** Analysis of forces.

Parameters of Force Measurements	Group 1	Group 2	% Diff Group 2 vs. Group 1	% Difference S9–10 vs. S1–2 in Group 1	% Difference S9–10 vs. S1–2 in Group 2
$F_{mean}^{right}$	3.23378	3.32861	2.932471	−33.036	−10.7484
$F_{mean}^{left}$	3.130066	3.361978	7.409167	−13.9833	−7.16814
$F_{std}^{right}$	1.602262	1.677908	4.721194	−44.5339	−4.89131
$F_{std}^{left}$	1.636178	1.664	1.700452	−38.0757	6.912041
$F_{max}^{right}$	7.440562	7.404284	−0.48757	−40.0544	−4.70138
$F_{max}^{left}$	7.207226	7.418431	2.930461	−22.9619	−0.15563
$F_{min}^{right}$	1.115157	1.093743	−1.92032	−8.27834	−7.12886
$F_{min}^{left}$	1.143257	1.101681	−3.63659	54.48436	−16.6559
$F_{int}^{right}$	292.9955	554.118	89.12169	−16.6674	−27.9686
$F_{int}^{left}$	264.0737	553.0317	109.4232	−7.45437	−25.5471
relative moment of $F_{max}^{right}$	−0.18384	−0.10031	−45.4335	172.9532	259.3863
relative moment of $F_{max}^{left}$	−0.25515	−0.15481	−39.3265	367.6416	17.76975
relative moment of $F_{min}^{right}$	−0.26997	−0.1258	−53.4028	−29.3991	289.4865
relative moment of $F_{min}^{left}$	−0.26446	−0.07727	−70.7802	220.8015	−42.983
maximal difference of consecutive extremums right	5.751664	5.336946	−7.2104	−38.65	−3.86
maximal difference of consecutive extremums left	5.4427	5.5412	1.8093	−33.601	6.1558
number of local maxima right	8.366667	10.29444	23.04117	−1.54639	0.530504
number of local maxima left	8.183333	9.933333	21.38493	1.11 × 10^−14^	−2.98913
number of local minima right	8.35	10.27222	23.02063	−2.57732	0.797872
number of local minima left	8.15	9.911111	21.60873	1.052632	−2.74725
position-related task time	8.944167	9.221111	3.096369	−5.62303	−16.1943
overall duration	17.73583	18.14944	2.332065	−5.31215	−16.4159

Abbreviations: S1–2: series 1 and 2; S9–10: series 9 and 10.

**Table 3:** Analysis of EEG.

Parameters of EMG Measurements	Group 1	Group 2	% Diff Group 2 vs. Group 1	% Difference S9–10 vs. S1–2 in Group 1	% Difference S9–10 vs. S1–2 in Group 2
$EMG_{MAV}^{back\_right}$	0.022175	0.03393	53.00936	−9.24482	−7.92041
$EMG_{MAV}^{back\_left}$	0.006357	0.032487	411.0173	−21.2933	−11.0977
$EMG_{MAV}^{chest\_right}$	0.015085	0.018502	22.65795	−36.619	10.87285
$EMG_{MAV}^{chest\_left}$	0.008021	0.018606	131.9509	24.61576	−10.82
$EMG_{MAV}^{arm\_right}$	0.029902	0.042155	40.9753	−9.45086	−16.501
$EMG_{MAV}^{arm\_left}$	0.029903	0.042155	40.9739	−9.45083	−16.5009

Abbreviations: S1–2: series 1 and 2; S9–10: series 9 and 10

## Abbreviation List

Abbreviation	Meaning
WMSD	Work-related Musculoskeletal Disorder
P&P	Pushing and Pulling
EMG	Electromyography
EEG	Electroencephalography
DASS	Depression Anxiety Stress Scales
VRSMAV	Verbal Rating ScaleMean Absolute Value
